# Microstructure and properties analysis of Ni60-based/WC composite coatings prepared by laser cladding

**DOI:** 10.1016/j.heliyon.2024.e24494

**Published:** 2024-01-11

**Authors:** Huang jiang, Zhu Zhikai, Shi Wenqing, Zhao Yang, Jiao Tianwen, Li Kaiyue

**Affiliations:** School of Electronics and Information Engineering, Guangdong Ocean University, Zhanjiang 524088, Guangdong, China

**Keywords:** Ni-based, laser cladding, WC particles, Corrosion resistance, Microhardness

## Abstract

In this study, Ni60-WCx coatings (x = 0, 2, 4, 6 %) on 316L stainless steel (316Lss) were prepared via laser cladding technology. We examined all specimens s for microstructure, phase composition, microhardness and electrochemistry using several characterization techniques. It shows that the microstructure of the Ni-based coatings can be changed with WC powder. When the WC ratio is 2 %, crystalline crystals and cellular crystals can be found in the coating. As the WC ratio increases, more cellular crystals and fewer spiny crystals appear in the coating. When the WC ratio changes to 6 %, only cellular crystals can be found in the coating. The microhardness resultsshow that the Ni-based overcoat with added WC has a better microhardness compared to the pure Ni coating, and its average value of the coating area reaches a maximum value of 822.8 HV at a WC ratio of 2 %. That is due to the addition of WC which can cause regime transition. In addition, the Ni-based coating has better corrosion properties due to its different microstructure. When the WC ratio is 2 %, the specimen possesses the maximum Ecorr and smaller icorr with the best corrosion resistance.

## 1 Introduction

Laser cladding, who has the superiorities of low dilution rate, strong metallurgical bonding with the substrate, and no environmental pollution, is widely used in aerospace, aviation, mechanical manufacturing [[1], [2], [3], [4]]. With the help of laser cladding, the mechanical and chemical properties of the coating can be effectively improved, such as high temperature oxidation resistance, microhardness, corrosion resistance, and laser cladding has become one of the most widely used surface modification methods.

316Lss shows good machinability, stable mechanical properties and high temperature oxidation resistance. 316Lss is widely used in bridge steel structure, building materials and other fields for its low cost and mature producing technology. Nevertheless, the microhardness and corrosion resistance of 316Lss are not outstanding, its microstructure and performance indicators can't adapt to the practical requirements of harsh working environments and biomaterials [[5], [6], [7]]. Laser cladding technology is used to enhance the comprehensive properties like corrosion resistance of 316Lss in the way of reconstructed the micro-structure. As we known, Ni-based self-fluxing powders have low melting point, good corrosion and wear resistance and self-lubricating effect, which is suitable to fabricate cladding coating of 316Lss. However, Ni-based powders have some disadvantages such as high porosity, imperfect microhardness, obvious thermal expansion, and so on [[8], [9], [10]]. In order to solve the disadvantages, researchers added certain amount of ceramics reinforcement particles into Ni-based claddings, because of their good corrosion and wear resistance, and high microhardness [[11], [12], [13], [14], [15]]. On the other hand, ceramic materials such as WC are widely used in metal-based cladding coatings own to their low thermal expansion coefficient, thermal stability, good wettability, and high microhardness. Zhou S F et al. [16] investigated the thermal stress mechanism of Ni-based cladding coatings by adding 20%-WC, they found that the residual stress, microstructure characteristics and porosities are three main influencing factors to determine its crack behaviors, and adjust the laser power process parameters with higher scanning speed can reduce the porosity. Qian Wang et al. [17] investigated Ni-WC gradient laser cladding coating with a mass fraction of 10%-WC, 30%-WC, 50%-WC, respectively. The coatings have no obvious defects, and the coating surface have good metallurgical bonding with the substrate. What's more, the surface layer has an average microhardness of 1053.5 HV, and noticeable wear resistance. Min Zeng et al. [18] have studied the corrosion resistance, microhardness and microstructure of Ni-WC laser cladding composite coating by adding 20%-WC. In addition the preparation method of WC has a significant effect on the micro-morphology and mechanical properties of Ni/WCcoatings. Ren et al. [19] prepared WC particles with 30 % Ni/WC composite coatings by agglomeration-sintering and sintering-crushing methods respectively. It showed that WC prepared by agglomeration-sintering could better enhance the microhardness and wear resistance of the coatings.

By looking at past studies, we can find that WC has a significant micorhardness enhancementand reduces the appearance of defects such as porosity. However, previous studies mainly focused on adding fixed content of WC to realize the improvement of Ni-based coating properties by changing the output laser energy. Alternatively, adding more than 10%-WC (large mass fraction) has great influence on the properties of the composite coatings. The effect of adding WC on the corrosion resistance of Ni-based coatings is also relatively scarce. Consequently, in this study, employing laser cladding technology, we select 316Lss as the substrate material, Ni60-WC_x_ as the coatings, where x is mass fraction of 0, 2, 4, and 6 %, respectively. Also, the morphology, microstructure, microhardness, corrosion resistance were studied.

## 2 Experimental

### 2.1 Materials

**Fig. 1:**
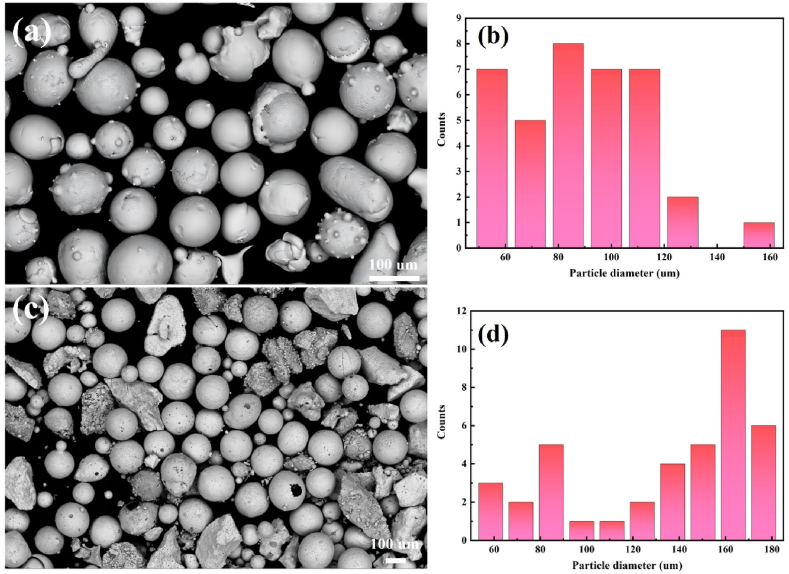
(a) Morphology of Ni60 powders; (b) spherical Ni60 powders distribution map; (c) Morphology of WC particle; (d) WC particle distribution map.

Before the experiment, we first prepared the experimental substrate. The 316Lss plate was cut to 50 mm × 50 mm × 2 mm. Then the plates were sanded with 800 grit sandpaper to achieve the removal of oxidized layer and impurities. Finally we cleaned it with alcohol. For the powder treatment, we first selected Ni60 and WC with a particle size range of 20–50 μm and mixed them for 2 h using a CZ0001 planetary ball mill. Finally, we dried them in the oven for 2 h until it was completely dry with a temperature of 100 °C. Fig. 1 (a) and (b) are morphology and distribution map of Ni60 powder, Fig. 1 (c) and (d) are morphology and distribution map of WC particles, respectively. WC particles are partly spherical and partly block. Table 1 lists the chemical composition of standard 316Lss and Ni60 alloys.

**Table 1:** Chemical composition of 316Lss and Ni60powder (mass fraction, %).

materials	Cr	Si	C	Mn	Ni	Fe
316Lss	16–18	1.5	≤0.03	2.0	10–14	Bal.
Ni60	14–19	3.5–5.0	0.5–1.0	0	Bal.	5.0–8.0

### 2.2 Preparation of the coatings

The equipment is a XL-F2000 fiber processing machine with a wavelength of 1064 nm, the maximum output power is 2000 W. With some former experiments and experience, we set the experiment process parameters as follows: the output power of 1000W, overlap ratio is 50 %, scanning speed is 300 mm/min, the defocus amount is 2 mm. After multi-pass laser cladding, we use wire cutting machine to make the specimen. Then we used 800, 1000, 1200, 2000, and 3000 grit sandpaper to meticulously polish the specimens, and finally we used a grinding machine and diamond polish to polish the specimens. The specimens were etched in the solution of aqua regia to analysis its surface quality, microstructure, composition and distribution by Nikon D3500 digital camera, XJL-302/302BD optical microscope (OM), TM4000plus scanning electron microscope (SEM). The microhardness test of the cladding and substrate was required by a MHVD-1000AT hardometer with a measuring load of 200 g and a load holding time of 10 s. The phase structures were examined by TD-5000 X-ray diffractometer (XRD). The diffraction angle ranges from 20° to 90°, and the scanning speed is 5°/min. The specimens of electrochemical corrosion were cut as 10mm × 10mm × 2 mm, the test surfaces were polished with sandpaper and diamond grinding pastes, and the non-test surfaces were sealed with epoxy, to make sure the exposed area equals 10 mm^2^. Before electrochemical measurement, the specimens were cleaned and washed by purified water, then immersed in 3.5 % NaCl solution for 1 h. After the previous preparations, the processed specimens were measured by CHI660E electrochemical workstation. For all the specimens we performed an open circuit voltage test for 30 min. The potentiodynamic polarization curves were given with a scanning range of −1.4 V to +0.6 V at room temperature under a scanning speed of 1 mV/s.

## 3 Results and discussion

### 3.1 Morphologies

**Fig. 2:**
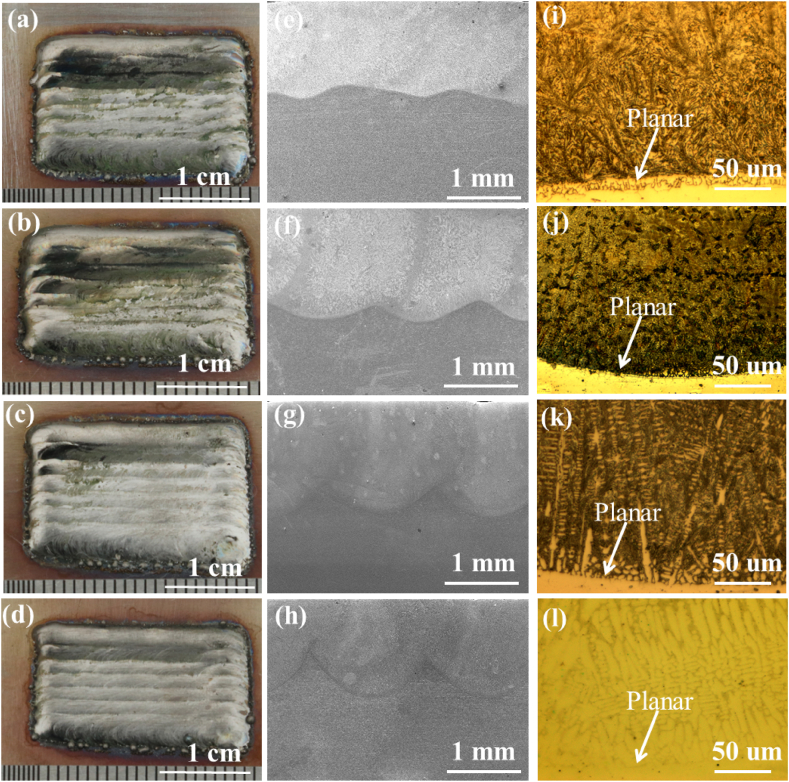
Morphology of Ni-based Composite Laser Cladding Coatings. (a)–(d) macroscopic morphology for different WC compositions, 0%-WC, 2 %, 4 %, and 6 %; (e)–(h) OM images depicting respective cross-sectional views: (i)–(l) SEM images of the respective cross-sections.

Fig. 2 are the Macrograph, OM and SEM images of Ni60-WCx (x = 0 %, 2 %, 4 % and 6 %) composite laser cladding coatings (Fig. 2 a-d) and their OM (Fig. 2 e-h) and SEM images (Fig. 2 i-l). As shown in Fig. 2 (a)–(d), all the specimens have smooth surface and present metallic luster without any defects. To observe the bonding characteristics of the cladding matrix with the substrate, the OM images of sectional regions of the specimens are shown in Fig. 2 (e)–(h). It shows that the metallurgical bonding is formed between the cladding coatings and substrates. Laser energy melts the composite powders, then thermal conduction occurs, the energy transfers from molten powders to the substrate. After the laser cladding progress and the specimens are rapidly cooled. The specimens are combined with the Ni60-WCx coatings and 316Lss substrate which produce a lower dilution rate and a good metallurgical bonding [20]. Fig. 2 (i)–(l) show the SEM of the specimens. The specimens with several mass fraction of WC show different morphologies of planar structures of the interface, which further proves the above conclusions [21]. The crystal structure changes from the interface to the coating surface, which mainly depends on the temperature gradient and solidification rate during the solidification of the molten pool [22].

### 3.2 Phase and microstructure

**Fig. 3:**
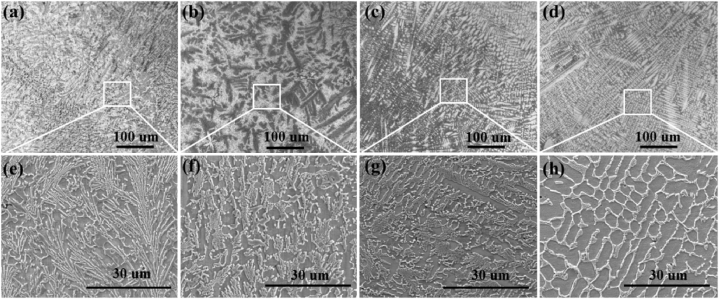
(a)–(d) The cross-sectional SEM of the coating in the middle region of all the coatings (e)–(h) are the partially enlarged views of (a)–(d), respectively.

The low and high-resolution SEM images of Ni60-WCx coatings of the microstructure are shown in Fig. 3. It shows from Fig. 3 (a)–(d) that the microstructure of Ni60-WCx coatings change with regular rules with the different mass ratios WC addition. Among these sample, the specimens with 0 % and 2 % WC show spicate-like structure in Fig. 3 (e)–(f). And when the WC mass ratios increase to 4 % and 6 %, the cellular crystals apear instead of spicate-like structure. After WC added, the mainly sructure transform from spicate-like crystals to cellular crystals, that means different mass ratios WC addition influence the microstructure of Ni60 coatings during laser cladding. In Fig. 3 (b)–(d), there are no WC particles can be found in the SEM images. This result means WC is completely molten and dissolved in the melt pool [23].In general, it shows that the microstructure of the coatings are changed with the increase of WC. When the 2 % WC added, the ratio of original spicate-like crystals reduced. While the mass fraction of WC increased to 6 % the coating presented cellular crystals instead of spicate-like crystals.

Table 2 listed the EDS analysis of the coatings, elements of C, O, Si, Cr, Mn, Fe, Ni, W are tested with atomic ratios. It is shown in Table 2, the content of W element is proportional to the addition of WC particles. What's more, W element can be analyzed in the coatings, that means WC is completed melted with Ni60. Due to the small atomic number of W, O, Si, Mn, their contents could not be analyzed exactly.

**Table 2:** EDS analysis of different points of the cladding coatings (atomic fraction, %).

Content	C	O	Si	Cr	Mn	Fe	Ni	W
0%-WC	9.11	3.00	4.77	13.62	0.27	23.88	44.88	0
2%-WC	9.95	0	6.49	9.82	0.80	20.31	51.88	0.55
4%-WC	10.03	1.52	0	47.69	0	30.95	8.59	1.22
6%-WC	32.61	0	0	9.61	0	25.79	30.78	1.21

**Fig. 4:**
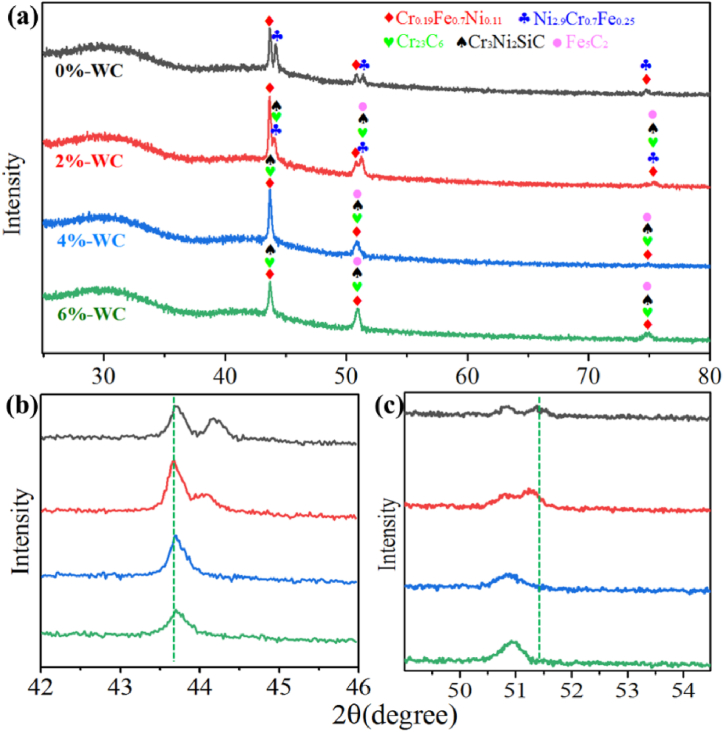
XRD result of the coatings.

The XRD patterns of the coatings and the details are shown in Fig. 4. In Fig. 4(a), the main peaks at 43°-45° and 50°-52° are lead to intermetallic compounds like Cr_0.19_Fe_0.7_Ni_0.11_ (JCPDS no.33-0397), Ni_2.9_Cr_0.7_Fe_0.25_ (JCPDS no.33-0945). When the ratio of WC increase to 4 % and 6 %, kinds of metallic carbides displayed, which lead to Cr_23_C_6_ (JCPDS no.35-0783), Cr_3_Ni_2_SiC (JCPDS no.17-0330) and Fe_5_C_2_ (JCPDS no.51-0997) [[24], [25], [26]]. The XRD patterns have been changed with the addition of WC, however, there is no typical peaks of WC showed in the XRD patterns. This is because the mass fraction of WC particles is low [27]. The XRD pattern of Ni60-WCx (x = 2 %) shows different trend compare with that of Ni60-WCx (x = 0 %). Cr_23_C_6_, Cr_3_Ni_2_SiC and Fe_5_C_2_ were formed after adding WC. And when the ratios of WC are 4 % and 6 %, the peaks lead to intermetallic compound of Ni_2.9_Cr_0.7_Fe_0.25_ are replaced to kinds of metal carbides.

Fig. 4(b)–(c) are the details of Ni60-WCx coatings in Fig. 4(a) at degree of 42°-46° and 49°-54°, the peak at 43.7° and 44.1° are mainly own to Cr_0.19_Fe_0.7_Ni_0.11_ and Ni_2.9_Cr_0.7_Fe_0.25_, when 2 % WC is added, the intensity of Ni_2.9_Cr_0.7_Fe_0.25_ (111) decrease obviously, and the peak of metal carbide appears. When the WC ratios change to 4 % and 6 %, the metal carbides appear instead of Ni_2.9_Cr_0.7_Fe_0.25_. The same condition is shown at the peak of 51.3°. The results demonstrate that the addition of WC can obtain faster nucleation rate of metal carbide than that of intermetallic compounds [28,29].

### 3.3 Microhardness

**Fig. 5:**
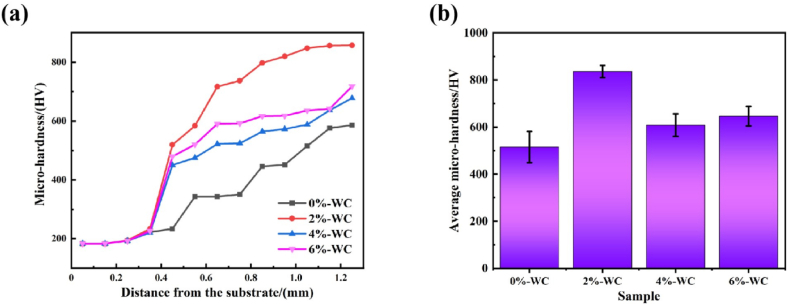
(a) Microhardness distribution ranging from substrate to the surface of the cladding coatings; (b) Average Microhardness of specimens' cladd**ing zone.**

Fig. 5 shows the microhardness in the cross-sectional specimens. In Fig. 5 (a), all Ni60-WCx coatings get higher microhardness than 316Lss substrate whose microhardness is about 188.1 HV. When x is 0 %, the average microhardness at heat-affected, bounding and cladding zone are 397.3, 515.5 and 515.1 HV, respectively. Fig. 5 (b) is the average microhardness of the coatings. When x is 2 %, 4 % and 6 %, the average microhardness at heat-affected zones can be 475.4, 383.6 and 393.6 HV, the average microhardness of bounding zones are 575.5, 586.4 and 626.1 HV; what's more, the average microhardness of their cladding zones are 835.9, 608 and 646 HV, respectively. Above all, when the WC ratio is 2 %, the Ni60-based coating can get highest microhardness, and the details can be shown in Table 3. The increase of WC content makes some WC particles melt, and the melted WC increase the precipitation of W, C and other elements, forming new nucleation cores and grain boundaries. Nucleation increases the solidification rate, resulting in the grain refinement strengthening [30,31].

**Table 3:** The average microhardness distribution of Ni-based cladding coatings (mass fraction, %).

Sample Microhardness (HV) Zone	substrate	Heat-affected zone	Bounding zone	Cladding zone
0%-WC	188.1	397.3	515.5	515.1
2%-WC	188.9	575.5	575.5	835.9
4%-WC	188.6	586.4	586.4	608
6%-WC	188.1	626.1	626.1	646

It is noteworthy that the microhardness of all the specimens with WC addition increased compared to the microhardness of the base material or the Ni60 coating without WC addition. Compared with 2%-WC, the microhardness of 4%-WC and 6%-WC decreased. This is due to the fact that with the addition of relatively excessive amounts of WC, most of the WC particles were dissolved in the melting-pool during the laser melting process, which reduced the microhardness of the final coatings [32]. However, as the WC content increases, the undissolved WC particles also increase relatively. This is why the microhardness of 6%-WC is slightly higher than that of 4%-WC. Therefore, adding a certain mass fraction of hard WC particles in the laser cladding process is an effective method to improve the hardness of the coating.

### 3.4 Corrosion resistance

**Fig. 6:**
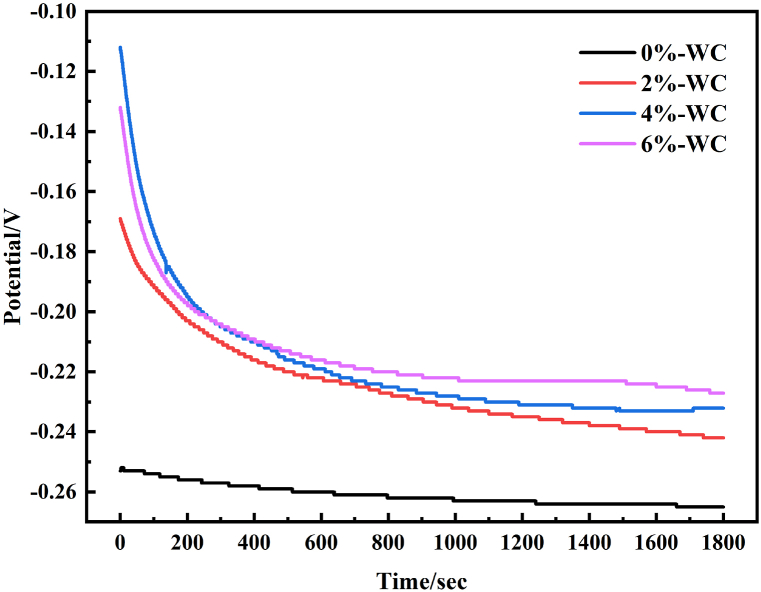
The Open-circuit potential of all specimens.

The open circuit potential (OCP) was measured in 3.5 % NaCl solution for 30 min, and the variation of OCP with time is shown in Fig. 6. OCP represents the corrosion tendency of the material; a more positive OCP means a lower surface electrochemical activity [33]. The results showed that all specimens stabilized after about 17 min, which means that a stable passivation film was formed in the coating [34]. Among them, the 4%-WC sample had the most positive OCP, which means that their electrochemical activity is higher.

**Fig. 7:**
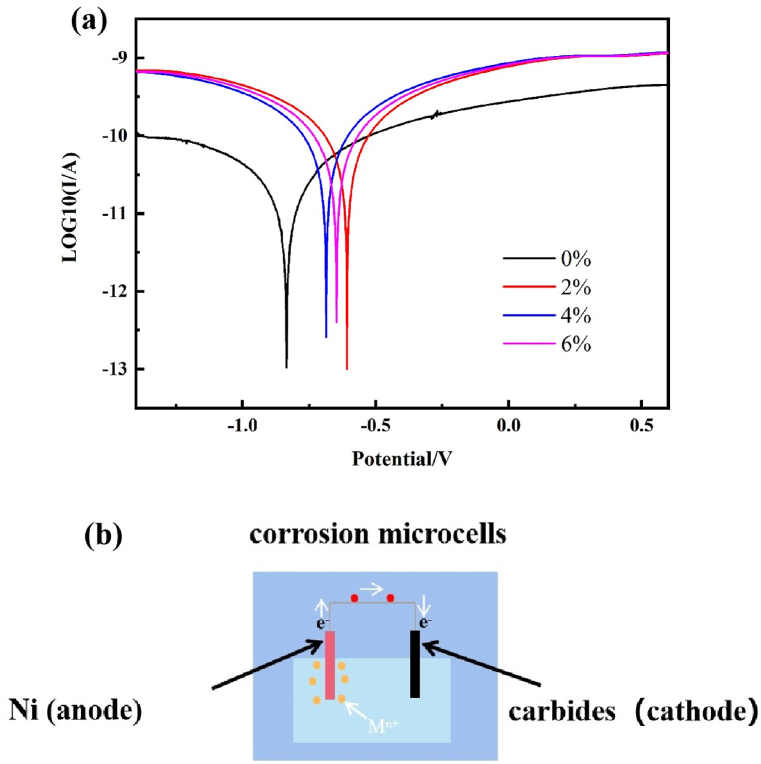
(a) Potentiodynamic polarization curves; (b) Primary Cell Mechanism Diagram.

Potentiodynamic polarization curves (PPCs) were used to evaluate the corrosion performance of Ni_60_-WC_x_ laser claded coatings in 3.5 % NaCl solution, and the results are shown in Fig. 7 (a). We obtained the self-corrosion potentials (E_corr_) and self-corrosion current densities (I_corr_) for all the specimens using Tafel extrapolation fitting with the structure shown in Table 4. As we know, Ecorr and Icorr are important parameters of corrosion resistance. In general, the stronger the corrosion resistance, the higher E_corr_ value, and the lower the corrosion thermodynamic tendency is; I_corr_ is the corrosion current, which reflects the generation speed of metal ions and is closely related to the corrosion rate [[35], [36], [37]]. From Table 4, we can understand that the values of I_corr_ are very close to each other when x is 2 %, 4 % and 6 %, so for this experiment we use E_corr_ to determine the superiority of corrosion resistance. The E_corr_ of coating immersed for X = 0 %, 2 %, 4 % and 6 % are −0.835 V, −0.608 V, −0.669 V and −0.666 V. The corrosion potential E_corr_ of X = 2 % coating shows better corrosion performance than that of other specimens, that is own to the appropriate addition of WC powders. As above, WC particles have obvious influence on the microstructure of the coating.When the WC ratio is 2 %, both spicate-like crystals and cellular crystals can be found in the coating. And the special structure is contributed to the enhancement of corrosion performance [38,39].

**Table 4:** The E_corr_ and I_corr_ of specimens.

Content	Ecorr (V/SCE)	Icorr (A/cm^2^)
0%-WC 2%-WC 4%-WC 6%-WC	−0.833 −0.609 −0.689 −0.649	8.636 × 10^−12^ 3.744 × 10^−11^ 3.626 × 10^−11^ 3.638 × 10^−11^

Notably, when the WC content exceeds 2 %, the corrosion resistance decreases. This is due to the fact that the excess WC is melted and diffused by the high temperature laser beam. Metal carbides are formed in the coating. There is an equilibrium potential difference between the metal carbide and Ni, forming a primary cell with the Ni as the anode and metal carbide as the cathode, as in Fig. 7 (b). According to the area impact of corrosion microcells, electrochemical corrosion is encouraged when cathode area and cathodic current density increase [40,41]. As WC increases, the metal carbide carbide increases and the area of cathode increases. Therefore the corrosion resistance decreases.

## 4 Conclusion

1. The micro-structure of Ni-based can be changed with the addition of WC powders. When the WC ratio is 2 %, both spicate-like crystals and cellular crystals can be found in the coating. More cellular crystals and less spicate-like crystals appear when the WC ratio changed to 4 %. When the WC ratio change to 6 %, only cellular crystals can be found in the coating.
2. The Ni-based cladding coatings with WC addition shows better microhardness compare with pure Ni coating. That is due to the addition of WC which can cause regime transition.
3. Moreover, the Ni-based coatings with WC show better corrosion performance because of their different micro-structure. When WC ratio is 2 %, the sample shows slowest corrosion rate.

Above all, the addition of WC powders can change the crystal growth mode in Ni-based cladding coatings effectively. We demonstrate that the regime transition is depended on the additive amount of WC. And the process is changed gradually.

## Data availability statement

No data was used for the research described in the article.

## CRediT authorship contribution statement

**Huang jiang:** Methodology, Conceptualization. **Zhu Zhikai:** Data curation. **Shi Wenqing:** Investigation, Formal analysis. **Zhao Yang:** Methodology. **Jiao Tianwen:** Resources, Project administration, Methodology. **Li Kaiyue:** Writing – review & editing, Visualization, Validation, Supervision, Software.

## Declaration of competing interest

The authors declare that they have no known competing financial interests or personal relationships that could have appeared to influence the work reported in this paper.
